# Factors Associated with Meeting Current Recommendation for Physical Activity in Scottish Adults with Diabetes

**DOI:** 10.3390/ijerph16203857

**Published:** 2019-10-12

**Authors:** Ahmad Salman, Kingsley Nnanna Ukwaja, Ahmad Alkhatib

**Affiliations:** 1 College of Health Sciences, QU Health, Qatar University, Doha 2713, Qatar; 2 Department of Health Sciences, University of York, York YO10 5DD, UK; 3 Department of Internal Medicine, Alex Ekwueme Federal University Teaching Hospital, Abakaliki PMB 102, Ebonyi State, Nigeria; 4 Institute of Sport Science, University of Taipei, Taipei 11153, Taiwan; 5 School of Health and Social Care, Teesside University, Middlesbrough TS1 3BX, UK

**Keywords:** physical activity, diabetes, health-related behavior

## Abstract

It remains unclear which factors are instrumental in meeting the recommended physical activity in people with diabetes. We, therefore, aimed to determine the sociodemographic, health-related behavior and clinical factors associated with meeting the recommended levels of physical activity in Scottish adults with diabetes. The study was based on the nationally-representative cross-sectional Scottish Health Surveys (2014–2017). The study participants included a sub-sample of 1259 adults (≥16 years old) with diabetes. Physical activity was evaluated using international guidelines. Overall, 34.1% of the subjects met the recommended levels of physical activity. Independent determinants of meeting the recommended levels of physical activity include male gender (odds ratio (OR) 1.47; 95% confidence interval (CI) 1.07–2.00) and being a non-smoker (OR 1.62; 95% CI 1.02–2.56). Furthermore, meeting the recommended physical activity levels decreased with age (OR 0.96; 95% CI 0.95–0.97), having a longstanding illness (OR 0.56; 95% CI 0.34–0.93) and body mass index (OR 0.94; 95% CI 0.92–0.97), but increased with higher fruit and vegetable intake (OR 1.16; 95% CI 1.07–1.25) and mental wellbeing (OR 1.04; 95% CI 1.02–1.06). Implementation of health promotion programs that target the identified determinants is needed to improve the recommended levels of physical activity among adults with diabetes.

## 1. Introduction

The prevalence of diabetes in individuals over 18 years of age increased from 4.7% in 1980 to 8.5% in 2014 with the number of affected adults increasing from 108 million to 422 million people [1,2,3]. A recent estimate in 2018 indicated there are over 500 million cases of type 2 diabetes globally, and that the burden of the disease is comparable between developed and developing countries [2]. The World Health Organization (WHO) estimated that diabetes was the seventh leading cause of death in 2016, with an estimated 1.6 million deaths being directly caused by diabetes and an additional 2.2 million deaths being attributable to elevated blood glucose [3]. Maintaining a healthy diet, adequate physical activity, body weight reduction and avoiding tobacco smoking or its cessation are lifestyle factors that could help lower the risk or delay the onset of diabetes [3]. Furthermore, self-management of diabetes is a complex process that involves regular assessment and monitoring of blood glucose, use of medications like insulin and other anti-diabetic medications, monitoring of carbohydrate intake, regular physical activity adherence, making adjustments to these factors (to maintain normal blood glucose levels), lowering the risk of complications and maintaining coping mechanisms to deal with the psychosocial aspects of living with diabetes [4,5].

Physical activity is essential in the management of diabetes. The American Diabetes Association and other international guidelines recommend at least 150 min per week of moderate to vigorous physical activity for individuals with type 2 diabetes [6]. Physical activity has been shown to help individuals with diabetes attain several management goals, such as improvement in their cardiorespiratory fitness, increased vigor, better glycemic control, lower insulin resistance, improvement in serum lipid profile, control of blood pressure and maintenance of weight loss [7]. Although the positive effects of regular physical activity are limited in individuals with type 1 diabetes, it has been shown to have beneficial effects on their blood glucose control and other health-related outcomes such as body weight monitoring, improvement in lipids, insulin sensitivity, self-confidence, improvement in mental wellbeing around diabetes and optimisation of long-term protection against cardiovascular diseases (CVDs) [8,9].

Despite the benefits of physical activity to individuals with diabetes, achieving the recommended level in this population could be a physical burden for affected persons leading them to abandon their exercise therapy [8]. This is because individuals with diabetes have lower physical performance threshold, show a reduced energy expenditure, have decreased step count and physical activity duration and lower cardiorespiratory fitness compared to healthy persons [8,9,10,11]. Furthermore, individuals with diabetes have lower muscle strength than those without the disease, and having a lower muscle strength is a determinant of having diabetic complications and disease progression in this population [11]. Therefore, diabetes and its complications may hinder the affected patients from undertaking physical activity [12]. In addition, several qualitative studies have evaluated attitudes and barriers to physical activity in individuals with diabetes [5,13,14,15]. Barriers to physical activity included: functional limitations due to diabetes or other medical conditions, “time, work, and lifestyle concerns”; difficulty changing well-established habits; social and personal factors; and environmental factors [5,13,14,15]. Only a few studies have quantitatively examined the factors associated with engaging in regular physical activity in adults with diabetes [16].

Analysis of government-supported national health surveys, such as the UK’s Scottish Health Survey, provides valuable information about the prevalence and associations of diabetes and physical activity [17]. Scotland shares the global burden of increased prevalence of diabetes and associated healthcare cost, with a crude prevalence of 5.5% in 2017 (compared to 5.1% in 2013) and one in 20 adults estimated to have diabetes [17,18,19,20]. Diabetes and associated chronic diseases in Scotland have also been linked with deprivation and socioeconomic status [17,18,20]. The prevalence of type 2 diabetes is 40% higher among people in the most deprived areas compared with those in the least deprived areas [18,20]. However, no data are available on whether deprivation explains lower physical activity levels, which is integral to diabetes prevention. Given the beneficial effects of regular physical activity for adults with diabetes, this study aimed to determine the sociodemographic, health-related behavior and clinical factors associated with meeting the recommended levels of physical activity in Scottish adults with diabetes. 

## 2. Materials and Methods 

### 2.1. Study Design and Participants

The Scottish Health Survey (SHeS) is a periodic population-based nationally-representative cross-sectional survey commissioned by the Scottish Government Health Directorates, which was designed to provide detailed information on the health status and well-being of the Scottish population [21]. The present study includes combined datasets covering the 2014–2017 period of surveys carried out by ScotCen Social Research, Edinburgh; MRC/CSO Social and Public Health Sciences Unit–University of Glasgow; the Centre for Population Health Sciences–University of Edinburgh; and the Public Health Nutrition Research Group–Aberdeen University. The data for the surveys from these years were combined because they shared a similar design and methods. Further details of the survey’s methods and questionnaire can be found on the SHeS website [17].

The participants of this study included all adults aged 16 years and over with a diagnosis of diabetes. Participants were classified as having diabetes if they reported a confirmed doctor diagnosis of either type 1 or type 2 diabetes. Gestational diabetes was excluded from the classification. Of the total 23,929 Scottish adults aged 16 years and over (10,976 men and 12,953 women) who participated in the 2014–2017 period, the analysis involved a total valid number of 1259 of adults with diabetes, which represents a prevalence of 5.26%. This observational study was reported following the guidelines of the Strengthening the Reporting of Observational Studies in Epidemiology (STROBE, Supplementary Materials).

### 2.2. Ethical Considerations

The Scottish Health Survey data were analyzed with the permission of the UK Data Service [21]. The survey data collection was approved by a central health ethics committee (Research committee for Wales REC reference number: 12/WA/0261).

### 2.3. Measures

The data collected from participants included whether or not they had any cardiovascular disease (CVD) and diabetes [17]. Data on physical activity levels, smoking habits, fruit and vegetable consumption and mental health were all collected by interviews using validated questionnaires, and other measures such as anthropometric measurements were also collected [17]. Participants were considered as having a long-term illness if they had any physical or mental health condition or illness lasting or likely to last, for twelve months or more [17]. Participants were classified as having ‘any CVD’ if they reported ever having any of the following conditions confirmed by a doctor: angina, heart attack, stroke, heart murmur, abnormal heart rhythm or ‘other heart trouble’ [17].

Physical activity interview module questions were based on the Allied Dunbar National Fitness Survey [17]. The interview questions consisted of four main types of physical activity: home-based activities (housework, gardening, building work or DIY); walking; sports and exercise; and activity at work. The information was collected based on time spent being active, the intensity of the activities undertaken, and the frequency with which activities are performed. Meeting the recommended level of physical activity was based on the UK adults’ guidelines of performing 150 min of moderate physical activity or 75 min of vigorous physical activity per week, or a comparable combination of both [18]. Fruit and vegetables interview module included questions on consumption of the following food types in the 24 h to midnight preceding the interview: vegetables (fresh, frozen or canned); salads; pulses; vegetables in composites (e.g., vegetable chilli); fruit (fresh, frozen or canned); dried fruit; fruit in composites (e.g., apple pie); and fresh fruit juice. Meeting the fruit and vegetable intake was based on meeting the 5/day daily intake recommendation [17]. Smoking status was classified as follows: current cigarette smoker, ex-regular cigarette smoker or never smoked cigarettes at all. Mental wellbeing was measured using the Warwick-Edinburgh Mental Wellbeing Scale (WEMWBS) questionnaire [22]. The area deprivation data are presented in Scottish Index of Multiple Deprivation (SIMD) quintiles [23]. It is based on 31 indicators in six individual domains (current income; employment; housing; health; education, skills and training; and geographical access to services and telecommunications). SIMD was reported in fifths with the first and second fifths being the least deprived, and the fourth and fifth being the most deprived.

### 2.4. Statistical Data Analysis

A descriptive analysis was carried out with categorical variables presented as proportions and continuous variables summarized using mean (SD: standard deviation). The main outcome variable was meeting the recommended level of physical activity among the study participants. The predictor variables include: economic/area deprivation data, having a long-term illness, having a CVD condition, dietary consumption of fruit and vegetables, smoking and mental wellbeing. A univariate analysis was first performed to determine the association between the outcome variable and potential predictor variables using the chi-square test. The independent t-test (or where appropriate ANOVA) was used to compare continuous variables. A multivariable logistic regression analysis was then performed to determine the independent predictors of meeting physical activity guidelines among the study participants. All data analysis was carried out using IBM Statistical Package and Services Solutions (SPSS) software version 25 (IBM Corp, Armonk, New York, USA). A *p* value ≤ 0.05 was considered statistically significant.

## 3. Results

Overall, 34.1% of the participants met the recommended levels of physical activity among the study participants. A chi-square test of independence was conducted between categorical variables and physical activity groups (Table 1). All expected cell frequencies were greater than five. There was a statistically significant association between all the categorical variables and physical activity groups.

An independent-samples t-test was run to determine any differences in continuous variables between physical activity (PA) groups. As seen in Table 2, age, body mass index (BMI), total portion of fruit and vegetables and WEMWBS score show a significant difference.

Binomial logistic regression was performed to ascertain sociodemographic and clinical factors associated with the likelihood of meeting recommended physical activity in Scottish adults with diabetes (Table 3). The area under the receiver operating characteristic (ROC) curve was 0.73, 95% CI (0.70, 0.77), which is an acceptable level of discrimination. The logistic regression model was statistically significant (χ^2^(14) = 153.68, *p* < 0.0001), explained 0.23% (Nagelkerke R^2^) of variance in meeting recommended physical activity and correctly classified 68.5% of cases. Sensitivity was 48.7%, specificity 81.8%, positive predictive value 64.2% and negative predictive value 70.4%. To assess the model for influential cases, Cook’s distance test and leverage values were computed but neither test produced unusually high values (*p* < 1.00 for all). The Hosmer–Lemeshow test in the final model was not statistically significant (*p* = 0.19), indicating that the model is not a poor fit.

Only nine variables were statistically significant: age, gender, longstanding illness, SIMD, had a CVD condition, smoking status, BMI, total portion of fruit and vegetables and WEMWBS score (Table 3).

Males had 1.47 times higher odds to meet recommended physical activity than females; 0.56 lower odds for those with a longstanding illness; 1.79 higher odds for those living in the three least deprived areas than among those in the most deprived areas; 0.54 lower odds for those with a CVD condition; 1.62 higher odds for those who never smoked at all or were an ex-regular smoker than current smoker.

Increasing the portion of fruit and vegetables and the WEMWBS score were associated with an increased likelihood of meeting recommended physical activity, but increasing age and BMI were associated with a reduction in the likelihood of meeting recommended physical activity.

## 4. Discussion

The findings of the current study showed that less than half of the participants in the present study met the current recommendations of physical activity, and a substantially higher proportion of men than women met the recommendations. The sociodemographic, health-related behaviour and clinical factors associated with meeting the recommended levels of physical activity in Scottish adults with diabetes were being a male, being in the middle quintile of deprivation rather than in the most deprived areas, being a non-smoker, having a higher fruit and vegetable intake as well as a higher mental wellbeing score. In addition, odds ratios for meeting the recommended levels of physical activity were lower with higher age, having a longstanding illness, having a CVD condition and having a higher BMI.

Concerns about the lack of physical activity amongst adults with diabetes have been repeatedly raised across different populations [24,25,26,27]. Those studies reported that approximately only 30% of individuals with diabetes have met the recommended physical activity guidelines, which is close to 34.1% found in the results of the present study. Among 13,608 US adults with diabetes (type 2 or type 1), it was reported that only 40.2% met the recommended physical activity guidelines (150 min/week moderate physical activity or 60 min/week vigorous physical activity), which was also used in the present study [27]. Another analysis of sub-cohort within the latter survey (998 adults with type 1 or type 2 diabetes) used a stricter threshold (90 min vigorous or 150 min/week moderate physical activity) and reported a lower physical activity adherence of 28.2% [28]. This suggests that irrespective of the diabetes prevalence (e.g., 5.5% in Scotland vs. 9.4% in US adults), reduced levels of physical activity require intervention [19,29].

In this study, we found that age was inversely associated with meeting the recommended levels of physical activity and that older adults with diabetes are at higher risk of reduced physical activity levels. This finding is supported by a lower adherence to physical activity guidelines of only 9.1% reported in older individuals with diabetes (average 73.4 years) [26], compared with a lower average age of 65.1 years (range 17–97 years) that corresponded to 34.1% physical activity adherence in this study. Similar age-related decline in physical activity levels amongst those with diabetes [30,31,32], though a few reported no association [33,34]. Reduced mortality risk among those with diabetes is associated with higher levels of physical activity [35]. The present study suggests the need for targeted approaches in older adults to increase their physical activity levels.

Gender was an independent predictor of meeting the recommended physical activity levels with 47% higher odds for diabetic Scottish men than for diabetic women of the 1259 individuals within the present study. This is consistent with lower physical activity levels in diabetic women than diabetic men reported in different populations, including Canadian (*n* = 244) [30], Omani (*n* = 305) [32], Jamaican (*n* = 194) [27] and US (*n* = 2977) [27] cohorts. Furthermore, only 28.8% of Scottish women with diabetes met physical activity guidelines, compared with 38.9% of their male counterparts. This suggests that a gender-dependent discrepancy in the adherence to physical activity guidelines among Scottish adults with diabetes may be addressed using global approaches, irrespective of region.

Deprivation and lower level of socioeconomic status are known causes of diabetes [36,37] Individuals living in the most deprived areas in Scotland have also demonstrated an increased prevalence of diabetes and associated chronic diseases [37,38,39,40]. The present study showed that >70% of Scottish adults with diabetes who belong to the most deprived quintile do not meet physical activity guidelines, compared with 60% of those who are at the least deprived quintile (Table 1). In addition, belonging to the middle deprivation area quintile was found to be associated with 79% higher odds of meeting the recommended levels of physical activity in the study populations. Our findings suggest that meeting physical activity guidelines requires addressing the challenges related to socioeconomic status in Scottish adults.

Our findings further support previous literature that demonstrates that avoiding tobacco smoking and having a higher fruit and vegetable consumption were independent predictors of meeting the recommended levels of physical activity in those with diabetes. In this study, we found that being “a never smoker” was associated with 62% higher odds of meeting the recommended levels of physical activity. Our finding is consistent with the results of Plotnikoff et al., who found that individuals with diabetes who were non-smokers were 2.75 times more likely to engage in physical activity compared with smokers [41]. Additionally, we found that having a higher fruit and vegetable consumption was associated with a 16% increase in the odds of meeting the recommended levels of physical activity per additional portion of fruit and vegetables consumed. Previous studies have shown that having a higher fruit and vegetable consumption was significantly related to good self-rated health and the tendency to maintain a healthy lifestyle, which includes physical activity [42,43]. Body mass index was another demographic variable that was found to negatively correlate with meeting the recommended level of physical activity in people with diabetes in the present study. This is in agreement with a previous study and suggests that diabetic patients with a high BMI need to be encouraged to sustain physical activity [41].

In this study, we found that the presence of medical conditions like having a longstanding illness or a CVD were independent predictors of not meeting the recommended levels of physical activity among patients with diabetes. This may be because these additional illnesses contribute additional disability to the affected patients making the management of their co-morbid illness difficult. Previous studies have found that having higher levels of disability or perceived disability (which could be due to diabetes-related complications or other chronic conditions), were negatively associated with physical activity in individuals with diabetes [41,44]. This study also found that having higher mental wellbeing (based on the WEMWBS score) was associated with 4% higher odds of meeting the recommended level of physical activity amongst the participants. Although the benefits appear marginal, this finding agrees with previous studies that demonstrated bidirectional relationships between physical activity and mental wellbeing [45,46], and supports the finding of another study that showed maintaining physical activity in patients with diabetes improved their mental wellbeing [47].

This cross-sectional study analyzed a large cohort of individuals with diabetes in the same region in Scotland, which provides valuable tools for designing effective targeted interventions. Future studies could use more objective measurement tools for physical activity such as accelerometers. This study used nine predictors within the binomial logistic regression, though future studies could also rely on additional predictors of meeting recommended levels of physical activity in adults with diabetes, such as ‘health workers’ advice to exercise’, “having no barriers to performing physical activity”, “having excellent general health”, “shorter duration of diabetes”, “decreased depressive symptoms”, “lack of time” and “lack of local facilities” which were not assessed in the present study [16,30,31,32,33,34,41].

## 5. Conclusions

In conclusion, this study has highlighted the low proportion of Scottish adults with diabetes meeting the recommended level of physical activity. In addition, the study found that being a male, being in the middle quintile of deprivation, being a non-smoker, having a higher fruit and vegetable intake, as well as better mental wellbeing, were independent predictors of meeting the recommended levels of physical activity, while having a higher age, longstanding illness, a CVD condition and a higher BMI were inversely related to meeting the recommended levels of physical activity in Scottish adults with diabetes. Findings from this study suggest that implementing health promotion programs that target these determinants are needed to improve the recommended levels of physical activity among Scottish adults with diabetes.

## Figures and Tables

**Table 1:** The proportion of participants meeting and not meeting the recommended levels of physical activity.

Characteristics (*N*)	Group (*N*)	Meeting PA Group %	Unmet PA Group %
Gender (1255) *	Male (669)	38.9%	61.1%
	Female (586)	28.8%	71.2%
Longstanding illness (1255) *	Yes (1144)	33%	67%
	No (111)	45.9%	54.1%
CVD (1253) *	Yes (499)	22%	78%
	No (754)	42.2%	57.8%
Smoking status (1254) *	Never smoked (565)	37.2%	62.8%
	Ex-smoker (470)	33.6%	66.4%
	Current smoker (219)	27.4%	72.6%
SIMD (1255) *	Least deprived (185)	38.4%	61.6%
	4th quintile (216)	38.4%	61.6%
	3rd quintile (296)	39.5%	60.5%
	2nd quintile (247)	27.5%	72.5%
	Most deprived (311)	28.9%	71.1%
BMI (912) *	Normal: 18.5 to less than 25 (94)	53.2%	46.8%
	Overweight: 25 to less than 30 (297)	42.8%	57.2%
	Obese I: 30 to less than 35 (297)	39.1%	60.9%
	Obese II: 35 to less than 40 (144)	33.3%	66.7%
	Obese: 40 or more (80)	26.3%	73.8%

BMI, body mass index; CVD, cardiovascular disease; *N*, number of participants; PA, physical activity; SIMD, Scottish Index of Multiple Deprivation; * *p* ≤ 0.05.

**Table 2:** Mean difference of continuous variables among groups.

Characteristics (*N*)	Meeting PA Group; *N*, Mean (SD)	Unmet PA Group; *N*, Mean (SD)
Age (1259) *	429, 61.30 (13.24)	826, 67.06 (12.89)
BMI (920) *	362, 30.70 (5.64)	555, 32.26 (6.10)
Total portion of fruit and vegetables (1258) *	429, 3.50 (2.33)	826, 2.78 (2.02)
WEMWBS score (1073)	390, 51.21 (8.75)	681, 46.78 (10.04)

BMI, body mass index; *N*, number of participants; PA, physical activity; SD, standard deviation; WEMWBS, Warwick-Edinburgh Mental Wellbeing Scale; * *p* ≤ 0.05.

**Table 3:** Binomial logistic regression predicting the likelihood of meeting recommended physical activity in Scottish adults with diabetes.

Characteristics	B	S.E.	Wald	df	*p*	OR	95% CI for OR	
							Lower	Upper
Age *	−0.04	0.01	33.68	1.00	0.00	0.96	0.95	0.97
Gender (male) *	0.38	0.16	5.84	1.00	0.02	1.47	1.07	2.00
Has longstanding illness *	−0.57	0.25	5.15	1.00	0.02	0.56	0.34	0.93
SIMD								
Least deprived	−0.05	0.26	0.04	1.00	0.84	0.95	0.57	1.58
4th quintile	−0.09	0.25	0.13	1.00	0.72	0.91	0.56	1.50
3rd quintile *	0.58	0.23	6.49	1.00	0.01	1.79	1.14	2.79
2nd quintile	−0.10	0.25	0.16	1.00	0.69	0.91	0.56	1.47
Most deprived as reference	−0.34		12.74	4.00	0.01	0.71		
Had CVD *	−0.61	0.17	12.45	1.00	0.00	0.54	0.39	0.76
Smoking status								
Never smoked at all *	0.48	0.23	4.25	1.00	0.04	1.62	1.02	2.56
Ex-regular smoker	0.38	0.24	2.39	1.00	0.12	1.46	0.90	2.35
Current smoker as reference	−0.86		4.25	2.00	0.12	0.42		
BMI *	−0.06	0.01	19.79	1.00	0.00	0.94	0.92	0.97
Portion of fruit and vegetables *	0.15	0.04	14.82	1.00	0.00	1.16	1.07	1.25
WEMWBS score *	0.04	0.01	20.62	1.00	0.00	1.04	1.02	1.06
Constant	1.62	0.76	4.53	1.00	0.03	5.04		

B, unstandardized regression coefficient; BMI, body mass index; CI, confidence interval for odds ratio; df, degrees of freedom; OR, odds ratio; S.E., standard error of the coefficient; SIMD, Scottish Index of Multiple Deprivation; WEMWBS, Warwick-Edinburgh Mental Wellbeing Scale; * *p* ≤ 0.05.

## Supplementary Materials

The following are available online at https://www.mdpi.com/1660-4601/16/20/3857/s1, S1: STROBE Statement—checklist of items that should be included in reports of observational studies.
